# Anticancer Activity of *Cynomorium coccineum*

**DOI:** 10.3390/cancers10100354

**Published:** 2018-09-26

**Authors:** Mouna Sdiri, Xiangmin Li, William W. Du, Safia El-Bok, Yi-Zhen Xie, Mossadok Ben-Attia, Burton B. Yang

**Affiliations:** 1 Sunnybrook Research Institute, 2075 Bayview Avenue, Toronto, ON M4N 3M5, Canada; sdiri.mouna88@gmail.com (M.S.); xiangmin227@163.com (X.L.); weidong.du@alum.utoronto.ca (W.W.D.); 2 Environment Biomonitoring Laboratory (LR01/ES14), Sciences Faculty of Bizerta, University of Carthage, Zarzouna 7021, Tunisia; benattia.mossadok@gmail.com; 3 Department of Laboratory Medicine and Pathobiology, University of Toronto, Toronto, ON M5S 1A8, Canada; 4 State Key Laboratory of Applied Microbiology Southern China, Guangdong Provincial Key Laboratory of Microbial Culture Collection and Application, Guangdong Institute of Microbiology, Guangzhou 510070, China; xieyizhen@126.com; 5 Laboratory of Biodiversity, Biotechnologies and Climate Change (LR11/ES09), Faculty of Sciences of Tunis, Tunis El-Manar University, Tunis 2092, Tunisia; safia1elbok@yahoo.fr; 6 Yuewei Edible Fungi Technology Co. Ltd., Guangzhou 510070, China

**Keywords:** *Cynomorium coccineum*, *Cynomorium songaricum*, MDA-MB-MB231, B16, Foxo3, breast cancer cells, tumorigenesis, mouse survival

## Abstract

The extensive applications of *Cynomorium* species and their rich bioactive secondary metabolites have inspired many pharmacological investigations. Previous research has been conducted to examine the biological activities and numerous interesting pharmaceutical activities have been reported. However, the antitumor activities of these species are unclear. To understand the potential anticancer activity, we screened *Cynomorium coccineum* and *Cynomorium songaricum* using three different extracts of each species. In this study, the selected extracts were evaluated for their ability to decrease survival rates of five different cancer cell lines. We compared the cytotoxicity of the three different extracts to the anticancer drug vinblastine and one of the most well-known medicinal mushrooms *Amaurederma rude*. We found that the water and alcohol extracts of *C. coccineum* at the very low concentrations possessed very high capacity in decreasing the cancer cells viability with a potential inhibition of tumorigenesis. Based on these primitive data, we subsequently tested the ethanol and the water extracts of *C. coccineum*, respectively in in vitro and in vivo assays. Cell cycle progression and induction of programmed cell death were investigated at both biological and molecular levels to understand the mechanism of the antitumor inhibitory action of the *C. coccineum*. The in vitro experiments showed that the treated cancer cells formed fewer and smaller colonies than the untreated cells. Cell cycle progression was inhibited, and the ethanol extract of *C.* *coccineum* at a low concentration induced accumulation of cells in the G1 phase. We also found that the *C. coccineum*’s extracts suppressed viability of two murine cancer cell lines. In the in vivo experiments, we injected mice with murine cancer cell line B16, followed by peritoneal injection of the water extract. The treatment prolonged mouse survival significantly. The tumors grew at a slower rate than the control. Down-regulation of c-myc expression appeared to be associated with these effects. Further investigation showed that treatment with *C.* *coccineum* induced the overexpression of the tumor suppressor Foxo3 and other molecules involved in inducing autophagy. These results showed that the *C. coccineum* extract exerts its antiproliferative activity through the induction of cell death pathway. Thus, the *Cynomorium* plants appear to be a promising source of new antineoplastic compounds.

## 1. Introduction

Cancer generates massive public burdens across the world, and the struggle to fight cancer and eventually find a cure is one of humankind’s greatest battles. Drugs to fight cancer, and many other diseases, are discovered and developed from many different sources, which include natural products like plants. There are many drugs today that are used to fight cancer that were originally obtained from natural sources [1,2]. A large problem today is that conventional chemotherapy comes with severe side effects, such as chemical toxicity to the host cells. Thus, research is underway to study possible chemotherapy drugs that are derived from plants [3]. *Cynomorium coccineum* is a traditional medicinal plant used extensively in the Arab world for its positive effect on fertility and immunomodulatory activities. *C. coccineum* and *C. songaricum*, belonging to the genus *Cynomorium,* are the only genus in the *Cynomoriaceae* family. *Cynomorium* plants are edible and nonphotosynthetic. The species *C. coccineum* is found in Northern Africa and the Mediterranean, and has different vernacular names depending on the region. It is called “tarthuth” according to the Arab world and “Maltese mushrooms” according to Europeans. *C. songaricum* is native to western Asia, widely collected, sold and used in the traditional Chinese medicine. This plant is considered a longevity food by the local Chinese people. Its local name, “Bu Lao Yao”, means “keeping people from becoming old” [4,5].

Over the past few decades, it has been proven that many compounds isolated from fungi and plants possess anticancer activity. The plant *Cynomorium* is one of them. Past biological and molecular pharmacologic studies of *Cynomorium* plants have demonstrated that these plants have the potential to help develop therapies that affect male reproductive function [6,7]. These plants also showed many other effects like anti-oxidative, antidiabetic, HIV-1-protease inhibitory, and immunity improving effects. This is due to the chemical constituents and bioactive compounds found in these plants such as polysaccharides, *triterpenoids*, *steroids*, *lignans*, *alkaloids*, and *flavonoids* [8,9,10,11,12,13].

In this study, we explored the anticancer activity of *Cynomorium* plants. We developed a series of experiments to illustrate the anticancer activity of *C. coccineum*. In vitro, we showed that the ethanol extract of *C. coccineum* inhibited cancer cell proliferation, survival, migration, invasion, and colony formation. In vivo, we found that the water extract of *C. coccineum* inhibited tumor growth and prolonged animal survival. These results provide a new possibility for the future development of *Cynomorium* plants as an effective, safe, medical drug for uptake by cancer patients. It also provides us insight to understand the molecular mechanism by which *C. coccineum* exerts its anticancer activity.

## 2. Materials and Methods

### 2.1. Plant Materials

*Cynomorium coccineum* is an edible plant found along the coasts of the Mediterranean Sea, depending on the bioclimate. *C. coccineum* can grow on different halophytic plants such as *Atriplex halimus*, *Obione portulacoides*, and *Inula crithmoides*. This plant was harvested from Bizerte, also known in English as Bizerta, the northernmost city in Africa. It is on a section of Mediterranean climate coastline close to Sardinia and Sicily. This plant was collected in Spring during the flowering period, precisely from a small coastal town in Bizerta, called Ghar al Milh, formerly known as PORTO FARINA, in the Sahel region of northeastern Tunisia. *C. coccineum* grows in subhumid and semi-arid coastal climates and saline soils in a large area along Mediterranean-type climate. This plant is nonphotosynthetic, which explains its color (brown-reddish for the absence of photosynthetic pigments). *C. coccineum* is a holo-parasite, characterized by a highly distinguishable form [8]. *C. coccineum* was first found and discovered by a Tunisian botanist researcher, Mounir Kasri, in 2011 in Ghar el Milh. The specimens were kept cool at about 4 °C in a portable refrigerator and transported to the laboratory within one hour. The aerial parts including the root weighed 1000 g and were 15–20 cm long and 4–7 cm wide. The specimens were cleaned and the residual earth removed then washed with distilled water. They were then cut into slices having a thickness of about 0.7 cm and dried at 50 °C. The dried *C. coccineum* were then powdered and stored at −20 °C. After drying, the weight of the dried material was about 400 g. *C. songaricum* was purchased in Toronto from a store selling Chinese medicinal materials. It is grown up in Gansu Province, China. This plant also possesses significant biological functions [6,14,15]. The samples were carefully handled as described with *C. coccineum and* stored at −20 °C for future use. 

### 2.2. Preparation of Cynomorium Plants

The extracts of the *Cynomorium* plants were prepared from the aerial part using boiling water. The dried aerial part of the *Cynomorium* plants were ground into powders that could be passed through a sieve with 60 pores per square inch. The powder (100 g each time) was incubated with hot water (1:10, *w*/*v*) at 100 °C for 2 h. After cooling down, the extract was filtered. The filtrate was evaporated. The partially dried material was transferred to a drier and maintained at 60 °C till completely dry. The dried powder was used to calculate yield. The procedure was repeated once. The dried powder was stored at −20 °C till use.

*Cynomorium* powder was extracted with 95% ethanol at a ratio of 1:5 (*w*/*v*). The extract was concentrated under a vacuum. Polysaccharide macromolecules are extracted through the “Hot water extraction and alcohol precipitation” method. This method allowed extraction of the *Cynomorium* plants material by aqueous medium. In brief, the grounded powder was soaked in water and subject to autoclaving at 120 °C for 20 min. It was allowed to cool down, and then followed by centrifugation at 1000 rpm at 4 °C for 10–15 min. Ethanol (95%) was added to the supernatant at the ratio of 1:5 (*w*/*v*). The mix was set at 4 °C to allow polysaccharide precipitation followed by centrifugation at 1000 rpm at 4 °C for 10–15 min. This step was repeated three times. The pellets were collected and dialyzed against water overnight. The contents were dried for later use. The extraction of *C. coccineum* was performed as described [5].

### 2.3. Cell Proliferation Assay

Cells were inoculated onto 24-well tissue culture plates at a density of 8 × 10^4^ cells/well in DMEM/RPMI 1640 containing 10% FBS with 100 U/mL penicillin/streptomycin. To examine the anticancer effect of the different extracts of the *C. coccineum* plant, we performed proliferation assays using three different human breast cancer cell lines MDA-MB-231(purchased from ATCC), MCF-7 (from ATCC), and MB468 (from ATCC), one mouse breast cancer cell 4T1, and one mouse melanoma cell line B16 (from ATCC). Four hours after cell inoculation, the extract of *Cynomorium* plants was added to the cultures at different concentrations (50, 75, 100, 150 μg/mL). Control was the cell cultures, to which the buffer was added. We also tested the effects of the extract on non-cancer cell lines NIH3T3 fibroblasts (from ATCC) and human lung cells BEAS-2B (from ATCC). The anticancer drug Vinblastine and the medium used to dissolve the polysaccharides served as controls. Some natural products that are known to possess anticancer activities were included for comparison, such as *Ganoderma lucidum* (100 µg/mL), *Amauroderma rude* (100 µg/mL), *Coriolus versicolor* (100 µg/mL), and Ergosterol (10 µg/mL). The cell cultures were maintained at 37 °C containing 5% CO_2_ for 48 h. The cells were harvested and mixed with trypan blue in a 1:1 ratio for exclusion staining. Dead cells were stained as blue, while living cells could not be stained by the dye due to the presence of the intact cell membrane. The number of living cells was counted as described [16]. Each experiment was repeated for at least three times for statistical analysis.

### 2.4. Cell Migration

Wound-healing assay was performed to analyze cell migration. In brief, 4 × 10^5^ cells were seeded onto 6-well dishes in DMEM medium containing 10% FBS and were maintained at 37 °C until they reached 90% confluence. The monolayer cultures were wounded by a sterile pipette tip to create a 1-mm cell-free path. The medium was removed, and the cultures were washed with PBS. The cultures were maintained in DMEM medium containing 10% FBS supplemented with or without *Cynomorium* at the concentrations indicated in the figures. Cell growth was inhibited by cell suppressor Mitomycin (2 μM). The cell cultures were photographed under a light microscope every 24 h. The distances between the wounding center and the front of the migrating cells were measured for statistical analysis.

### 2.5. Cell Invasion

Cell invasion experiment was performed as described [17]. Briefly, the trans-well inserts were loaded with 80 μL of Matrigel (1:8 dilution) and incubated at 37 °C for 30 min. This allowed a gel-bed to form. The inserts were then placed onto the wells of a 24-well plate, to which 500 μL of medium containing 10% FBS had been added. MDA-MB-231 cells (1 × 10^4^) in 100 μL serum-free medium containing different concentrations of *Cynomorium* were gently loaded on top of each gel bed. The cultures were incubated at 37 °C for 36 h. The trans-well inserts were then fixed with cool methanol for 15 min, followed by staining with Coomassie Brilliant blue. The upper Matrigel layer and cells were removed and the wells were cleaned. Cells that invaded through the Matrigel and spread onto the lower surface of the inserts were photographed under a light microscope. The stained cells were counted from representative fields for quantification.

### 2.6. Cell Survival

To determine cell viability, 1 × 10^5^ cells were cultured in 10% FBS containing DMEM in culture dishes. The cultures were maintained at 37 °C for 12 h. The culture medium was then removed, and the cultures were washed with PBS, followed by addition of serum-free DMEM with or without *Cynomorium* at the concentrations indicated in each figure. The cultures were maintained at 37 °C for 2 or 4 days. The cells were harvested, and cell number was counted by using trypan blue staining.

### 2.7. Cell Cycle Analysis

Cell cycle was analyzed by means of the propidium iodide staining procedure, previously reported in [16]. Changes in DNA content during cell cycle progression were measured with flow cytometry. MDA-MB-231 cells were seeded at the density of 5 × 10^6^ cells/well, with 2 mL culture medium containing 10% FBS. Four hours after cell inoculation, alcohol extract of *C. coccineum* at the concentration of 50 µg/mL was added. After 48 h incubation, the cells were harvested and washed twice with PBS, fixed with ice-cold 70% ethanol at 4 for 1 h and stained with propidium iodide solution in PBS. Cytofluorimetric acquisitions and analysis of DNA contents were performed by flow cytometry (BD Biosciences, San Jose, CA, USA).

### 2.8. Colony Formation

Colony formation was performed as described [18]. Briefly, 0.66% agarose gel was loaded on each well of the 6-well tissue culture plates. After solidification, MDA-MB-231 cells (1 × 10^3^ cells/well) and ethanol extract of *Cynomorium* plants (at the final concentrations of 0, 50, 100 μg/mL) were mixed with low melting agarose in DMEM supplemented with 10% FBS to obtain a final concentration of 0.3% agarose. The mixtures were plated on the top of the 0.66% agarose gel. Colony formation was monitored frequently. Twenty days after cell inoculation, the colonies were examined under a light microscope. For colony staining, the colony-containing agarose gel was prefixed with 80% methanol for 30 min, followed by staining with 0.25% Coomassie Blue for 2–4 h, until a uniform blue color was obtained. The gel was distained with 5% methanol, followed by washing in 10% acetic acid until background was clear. Colonies were photographed under a light microscope.

### 2.9. Western Blot

After being treated with the alcohol extract of *C. coccineum*, cells were lysed by the lysis buffer containing protease inhibitor (MilliporeSigma, Burlington, MA, USA) on ice for 30 min, followed by centrifugation to obtain clear cell lysates. Protein concentrations were determined by Bio-Rd Dc protein assay. Samples with equal amounts of proteins were subject to separation by SDS-PAGE and transferred onto a nitrocellulose membrane (Bio-Rd, Hercules, CA, USA). After blocking with 5% milk, the membrane was incubated with primary antibodies at 4 °C overnight. Next day, the membrane was washed three times. The membrane was then incubated with secondary antibody for 2 h and washed three times again. The membrane was developed with an ECL kit (MilliporeSigma, Burlington, MA, USA) and visualized using the Kodak Image Station 4000R (Kodak, Rochester, NY, USA) as described [19].

### 2.10. Real-Time PCR

Total RNA was extracted using Total RNA Mini kit as described [20]. The concentrations of RNAs were measured by NanoDrop 2000c UV-VIS Spectrophotometer (Thermo Scientific, Waltham, MA, USA) under 260 nm. Equal amounts of RNA were subject to reverse transcription using SuperScript III First-Strand Synthesis System (Invitrogen, Carlsbad, CA, USA) to obtain cDNAs. The cDNAs were diluted (1:5 dilutions) and used as templates for PCR using a SYBR Green PCR Kit (QIAGEN). Real-time PCR was performed for 40 cycles as follows: denaturation at 95 °C for 30 s, annealing at 59 °C for 30 s, and elongation at 72 °C for 30 s (ABI PRISM 7700 Sequence Detection System, Thermo Scientific, Waltham, MA, USA). The mRNA levels were analyzed using U6 as an internal control. The assay was performed in triplicate [18,21]. The primers for Foxo3 mRNA were 27.46.hu.Foxo3-F (5′gttcgctggccgcacgtcttc) and 27.48.hu.Foxo3-R (5′ggagggacgtggacgccgcga). The primers used for U6 were 19-19-Hu-U6RNA-F (5′caccgtgctcgcttcggcagcacatatac) and 19-20-Hu-U6RNA-R (5′accgtgcaccggcgtataaacgtggtgta).

### 2.11. Animal Survival Experiment

Four-week old mice were purchased from Charles River. We used a technique established in our laboratory previously [22,23]. The animals were kept in the Animal Core Facility of Sunnybrook Research Institute for one week before use. The mice were randomly divided into 2 groups. Twenty mice were injected intraperitoneally with tumor cells B16 (1 × 10^4^ cells/mouse). Three days after tumor cell injection and until palpable tumor formation, *C. coccineum* was injected intraperitoneally at a dose of 50 mg/kg mouse. The concentration of *C. coccineum* extract we routinely obtained was 100 mg/mL dissolved in DMSO. To have the concentration of 50 mg/kg mouse, a mouse received 1 mg *C. coccineum*. We diluted the initial product 10 times to obtain 10 mg/mL using distilled water. Thus, 100 µL was needed per mouse. This was repeated every other day for up to 20 days. The control group was mice injected with DMSO diluted 10 times in distilled water at the same volume and on the same schedule. All animal experiments were conducted according to the guideline approved by the Animal Care Committee at Sunnybrook Research Institute (ethic code 2017-076).

## 3. Results and Discussion

### 3.1. Cynomorium Coccineum Induces Cancer Cell Death by Modulating Cell Cycle Progression

*Cynomorium coccineum* has long been known as a medicinal tonic, which has been documented in different cultures. For instance, the plant is regarded as a remedy for various diseases and has been known to be used as an antihaemorrhoidal agent, a spermatogenesis stimulating agent, and an aphrodisiac. Additionally, the plant has been used as a tonic and shown antivomitive and hypotensive effects [9,24,25]. Some of the effects have been confirmed in studies using animal models [24,26]. The “fungus” plants were also used as a food, especially during periods of famine. This may be due to its relatively high content of oils rich in essential fatty acids, which are known to help people to survive [27,28]. The composition and lipid profile of fixed oil from dried stems of *C. coccineum* have been reported [27]. *C. coccineum* contains many active ingredients including anthocyanic glycosides, triterpenoid saponins, and lignans. It is known that *Cynomorium coccineum var. coccineum* from Sardinia contains *gallic acid and cyanidin-3-O-glucoside* as the main constituents [29].

**Figure 1:**
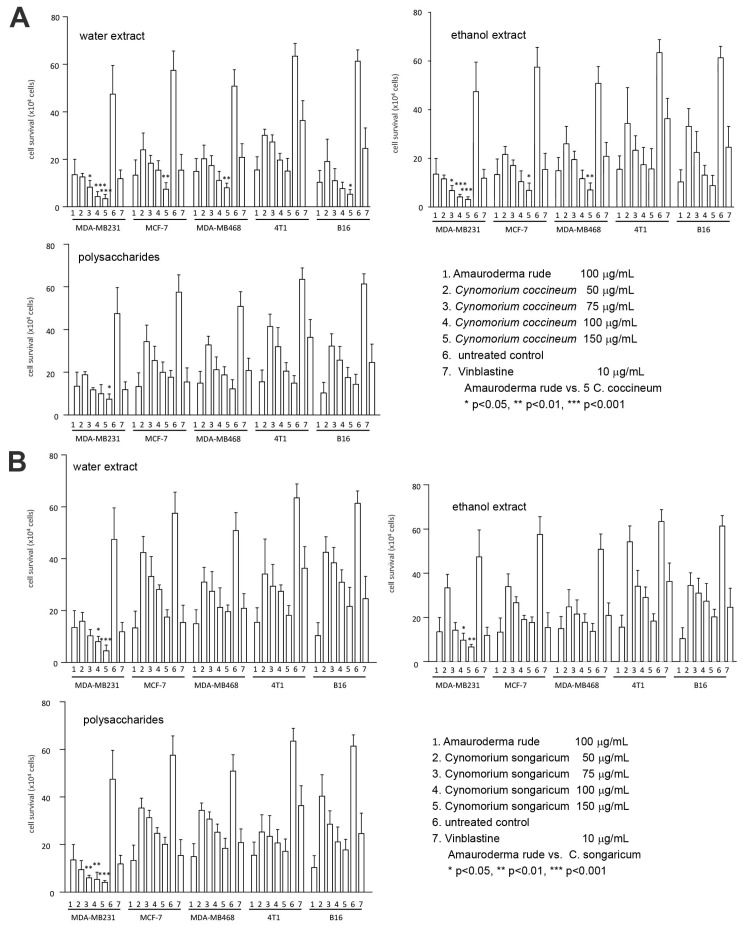
Comparison of bioactive components on cancer cell survival: (**A**) Cancer cell lines MDA-MB-231, MCF-7, MDA-MB-468, 4T1, and B16 were cultured and treated with or without *Amauroderma rude*, *C. coccineum*, and Vinblastine at the concentrations indicated. Cell viability was determined. (**B**) The cancer cell lines were cultured and treated with or without *Amauroderma rude*, *C. songaricum*, and Vinblastine at the concentrations indicated, followed by cell viability determination.

In this study, we first dried the plant and isolated the biologically active components by hot water extraction. We also prepared an alcohol extract and polysaccharide extract. The anticancer effects of these three extracts were examined on a number of cancer cell lines. We found that the preparations from all three isolation methods were powerful in the induction of cancer cell death (Figure 1A). Using the same approaches, we also isolated the water extract, alcohol extract, and polysaccharides from *C. songaricum*. Their bioactivities were found similar to *C. coccineum* in the induction of cancer cell death (Figure 1B).

**Figure 2:**
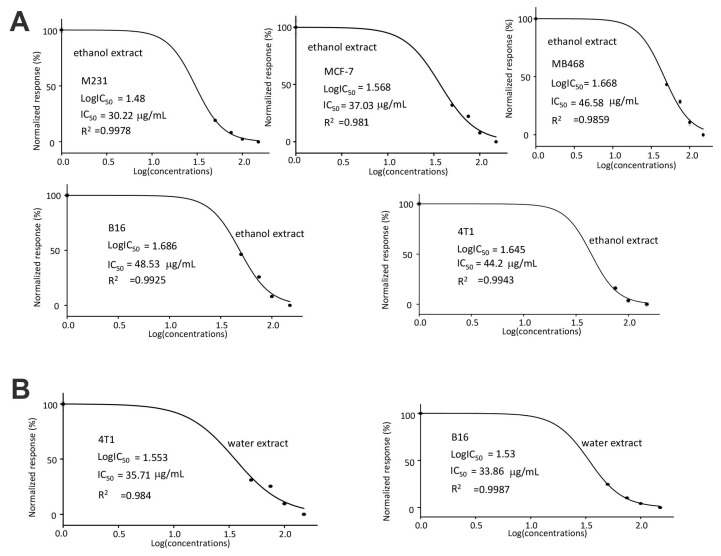
(**A**) IC_50_ values were calculated for MDA-MB-231, MCF-7, MDA-MB-468, B16, and 4T1 after treatment with the ethanol extract of *C. coccineum*. It showed that MDA-MB-231 cells were the most sensitive cell line to the ethanol extract with an IC_50_ of 30.22 µg/mL. (**B**) IC_50_ values were calculated for both B16 and 4T1 after treatment with the water extract of *C. coccineum.* The B16 cells were more sensitive to the water extract than 4T1 cells. (33.86 ± 1.7942 µg/mL).

In the half maximal inhibitory concentration (IC_50_) measurements, we found that the human breast cancer cell line MDA-MB-231 was most sensitive to the ethanol extract of *C. coccineum* (with the IC_50_ of 30.33 μg/mL, Figure 2A). In the mouse cell lines, B16 cells appeared to be more sensitive to the water extract of *C. coccineum* (with the IC_50_ of 33.86 μg/mL) than 4T1 cells that had an IC_50_ of 35.71 μg/mL (Figure 2B).

**Figure 3:**
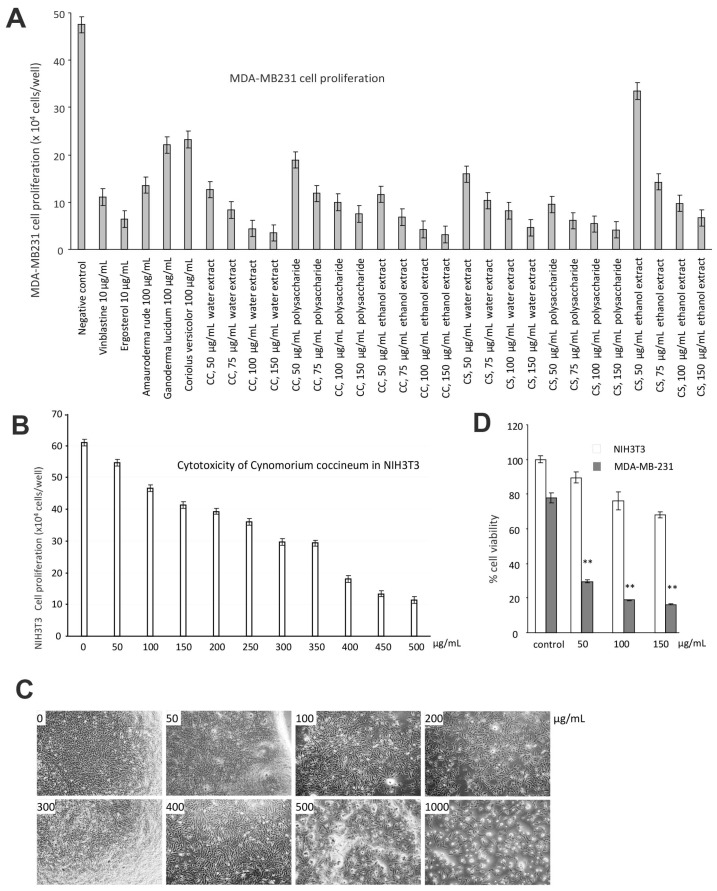
*C. coccineum* inhibits cancer cell proliferation: (**A**) MDA-MB-231 cells were cultured in tissue culture plates to subconfluency, to which the extracts of *C. coccineum* and *C. songaricum* were added at the concentrations as indicated. Vinblastine, ergosterol, extracts of *Ganoderma lucidum*, *Amauroderma rude*, and *Coriolus versicolor* were used as controls. The negative control contained the buffer alone. The extracts of *Cynomorium* plants were the most effective agents in inducing cancer cell death. (**B**) The effects of *C. coccineum* on the non-cancer cell line NIH3T3 fibroblasts were tested. Very high concentrations of *C. coccineum* were needed to induce cell death. (**C**) Typical photos of NIH3T3 fibroblasts treated with *C. coccineum* are shown. (**D**) The effects of *C. coccineum* are compared in NIH3T3 fibroblasts and MDA-MB-231 cells. *C. coccineum* possesses significantly stronger activity in inducing cell death in MAD-MB-231 cells relative to NIH3T3 fibroblasts. ** *p* < 0.01.

We thus used the human breast cancer cell line MDA-MB-231 for further study. Untreated MDA-MB-231 cells cultured in DMEM containing 10% FBS were used as a negative control. MDA-MB-231 cells treated with the anticancer drug Vinblastine served as a positive control. We have previously demonstrated that *Ganoderma lucidum*, *Amauroderma rude*, *Coriolus versicolor*, and Ergosterol possess anticancer effects [30,31,32,33,34]. Thus, these products were included in our assay as controls. We found that *Cynomorium* plants were the most effective agents in inducing cancer cell death (Figure 3A). We found that the extracts obtained from water and ethanol incubation were equally effective on inducing cancer cell death. We also noted that the ethanol extract was more stable in activity than the water extract. We thus used the ethanol extract of *C. coccineum* in our further experiments. Since the assays were performed in the serum-free culture medium which is considered as a stressful environment, addition of ethanol extract of *C. coccineum* at the concentrations of 50 or 100 µg/mL significantly reduced cancer cell viability as compared with the buffer control. It is previously reported that *Amauroderma rude* is a well-known medicinal mushroom which exerted a very strong activity in inducing cancer cell death [30,34,35]. Our study showed that *Cynomorium* possessed even stronger anticancer activity. Recently, it was shown that the water extract of *C. coccineum* possesses antioxidant activities [36]. In addition, different components from this species also exert antimicrobial and antityrosinase activities [37].

We tested the specificity of the ethanol extract of *C. coccineum* by using a noncancer cell line, NIH3T3 fibroblasts. Very high concentrations of *C. coccineum* were needed to induce cell death in these cells (Figure 3B). Even at higher concentrations, the ethanol extract of *C. coccineum* appeared to inhibit the growth of NIH3T3 fibroblasts, but there was a lesser sign of cell death (cell fragmentation) that could be seen (Figure 3C). We compared the effects of ethanol extract of *C. coccineum* on both groups of cells, cancer and noncancer. It showed that at the concentrations of 100 µg/mL, the ethanol extract of *C. coccineum* displayed significantly stronger activity on inducing the death of the cancer cells, compared to the noncancer cells (Figure 3D).

**Figure 4:**
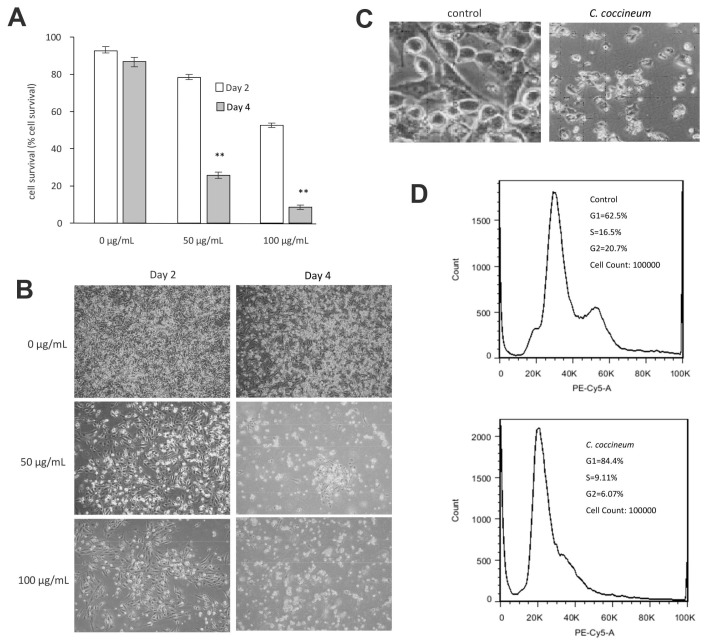
*C. coccineum* decreases cancer cell viability: (**A**) MDA-MB-231 cells were cultured in tissue culture plates to subconfluency in serum-free DMEM, to which the ethanol extract of *C. coccineum* was added at the concentrations as indicated. The cultures were maintained for 2 or 4 days. Addition of ethanol extract at the concentrations of 50 or 100 µg/mL significantly induced cell death as compared with the buffer control. (**B**) Typical photos showing detachment of cells from the plates treated with *C. coccineum*. (**C**) The extract of *C. coccineum* induced cancer cell fragmentation. (**D**) *C. coccineum* inhibits cancer cell cycle progression. We measured the cell cycle progression of the cells treated with the ethanol extract of *C. coccineum* (50 µg/mL). The treated cells had 84.4% of cells arrested in the G1 phase compared with un-treated cells having 62.5% in G1 phase. ** *p* < 0.01.

We found that ninety percent of cancer cells died when they were treated with 100 μg/mL of the ethanol extract of *C. coccineum.* Our results suggest that the polysaccharides are not the major active component in the medicinal plant possessing anticancer activity. The non-polysaccharide fraction may play an essential role in inducing cancer cell death. Thus, we used the ethanol extract in our studies. The addition of ethanol extract at the concentrations of 50 or 100 µg/mL significantly induced cell death as compared with the buffer control (Figure 4A). These cells initially detached from the tissue culture plates and then died (Figure 4B). Morphologically, the detached cells appeared fragmented and as typically apoptotic cells (Figure 4C).

We analyzed cell cycle progression by PI staining using flow cytometry. Cell cycle analysis revealed an increased population in G1 phase after treatment with *C. coccineum* (84% vs. 62.5%), where it showed a decrease in S and G2 phase (9.11% and 6.07% vs. 16.5% and 20.7%, respectively), compared with untreated cells (Figure 4D). The assay showed that the ethanol extract of *C. coccineum* at a low concentration of 50 μg/mL was sufficient to inhibit cell cycle progression of MDA-MB-231 cells. It showed that 84.4% of the treated cells were arrested in the G1 phase, compared with untreated cells that had 61.5% in the G1 phase.

### 3.2. Cynomorium Coccineum Inhibits Cancer Cell Migration, Invasion, and Colony Formation

**Figure 5:**
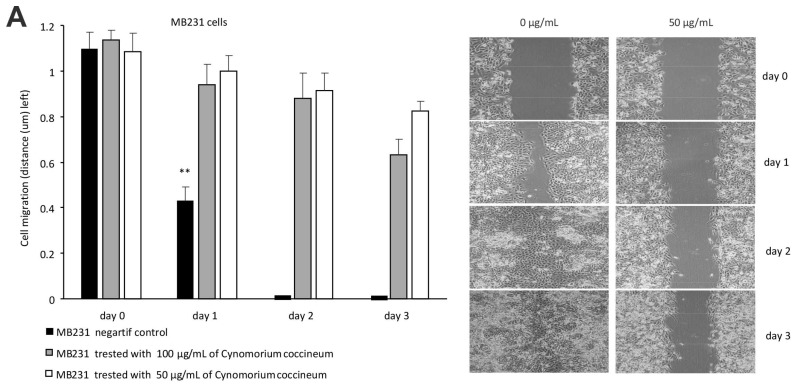

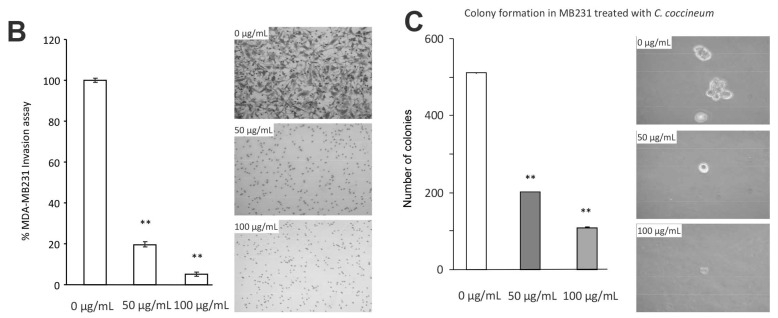
*C. coccineum* inhibits cancer cell migration, invasion, and colony formation: (**A**) Left, MDA-MB-231 cells were cultured in tissue culture plates to subconfluency for wound healing assay. Addition of the ethanol extracts of *C. coccineum* to the wounded cultures inhibited the healing process at 50 or 100 µg/mL of the extract. Right, typical photos of the cultures during the wound healing process. (**B**) Left, in a Matrigel invasion assay, we found that addition the extract of *C. coccineum* significantly inhibited MDA-MB-231 cell invasion at the minimum concentration of 50 μg/mL extract. Right, in the extract-treated cultures, some cells could invade through the Matrigel, but could not spread. (**C**) Left, in colony formation assay, MDA-MB-231 cells formed less (left) and smaller (right) colonies when the cultures were treated with *C. coccineum.* ** *p* < 0.01.

We tested the effect of the ethanol extract of *C. coccineum* on cell migration in a wound healing assay. In the control, the wound gap was completely healed 2 days after wounding. Addition of the *C. coccineum* ethanol extracts (50 or 100 µg/mL) inhibited the wound healing process (Figure 5A, left). Since cell proliferation was inhibited by the addition of the cell suppressor mitomycin (2 μM), the process fully relied on cell migration. In the cell migration assay, the culture medium contained 10% FBS. Thus, the cells stayed in healthy conditions (Figure 5A, right). The effect of *C. coccineum* on inhibiting cell migration was dramatic.

In a Matrigel invasion assay, we found that the ethanol extract of *C. coccineum* displayed a significant inhibitory effect on MDA-MB-231 cell invasion at the minimum concentration of 50 μg/mL of extract (Figure 5B, left). In the extract-treated cultures, some cells could invade through the Matrigel, but could not spread onto the lower surface of the inserts (Figure 5B, right). Again, the invasion assay was performed in the culture medium containing 10% FBS.

We further tested the effect of the ethanol extract of *C. coccineum* on colony formation of MDA-MB-231 cells. The experiment showed that at a minimum concentration of 50 μg/mL, the ethanol extract of *C. coccineum* significantly inhibited colony formation in both the number (Figure 5C, left) and size (Figure 5C, right) of colonies. Our results suggest that small molecules that were extracted by ethanol incubation were indeed responsible for the potent effects that the extracts had on inhibiting cell migration, invasion, and colony formation.

### 3.3. Cynomorium Coccineum Treatment Prolonged Animal Survival

**Figure 6:**
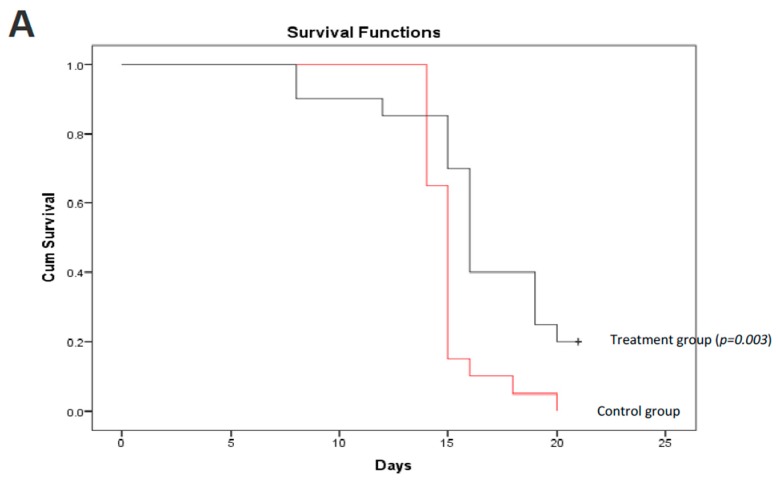

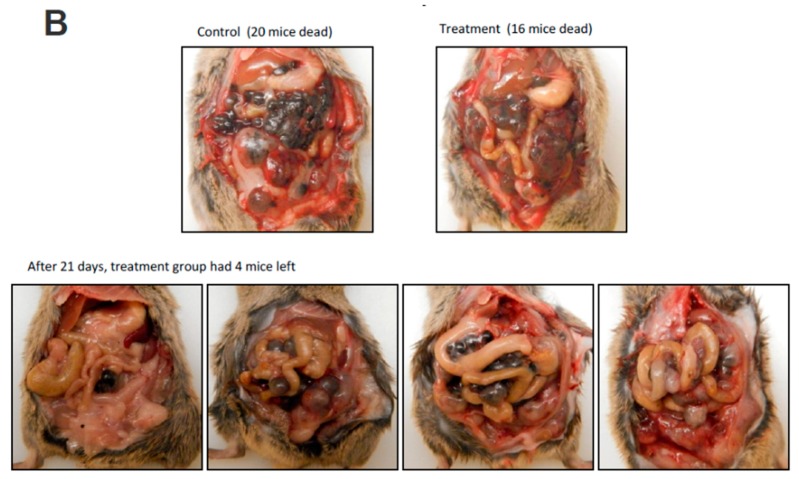
*C. coccineum* treatment prolonged animal survival. C57BL mice were injected with B16 tumor cells. Three days after cell injection, *C. coccineum* water extract was injected every other day at the concentration of 50 mg/kg mouse. Tumor development and mouse viability were monitored every other day. On day 21, the mice treated with *C. coccineum* displayed a significantly longer viability than the control. Upper, Kaplan–Meier analysis of *C. coccineum* displayed survivor curves; Lower, photos showing tumors developed in the control but less in the *C. coccineum*-treated mice.

We further validated the anticancer effect of *C. coccineum* in vivo. In this assay, we adapted a mouse model of tumor formation to test the extract of *C. coccineum.* Strain C57BL mice were injected with B16 cells. B16 cells appeared to be more sensitive to the water extract compared to the ethanol extract of *C. coccineum* (Figure 2). We tested the inhibitory effect of the water extract of *C. coccineum* on tumor growth. Three days after the tumor cell injection, *C. coccineum* water extract was injected every other day at the concentration of 50 mg/kg mouse. On day 21, we found that the mice treated with the water extract of *C. coccineum* displayed a significantly longer survival time than the control mice, according to the Kaplan–Meier analysis of survivor curves (Figure 6A). The mean survival time of control groups ranged from 1 to 2 weeks, while the mean time of the treated groups ranged from 1 to 3 weeks. After 21 days, four mice from the *C. coccineum* water extract treated group were still alive (Figure 6B). This data suggests that the water extract of *C. coccineum* had anticancer effects in vivo and could prolong survival rates of tumor-bearing animals, leading to a longer lifespan. This may be of clinical significance.

### 3.4. Regulation of Signal Protein Expression by Cynomorium Coccineum

We examined whether *C. coccineum* extract induced cancer cell apoptosis by the upregulation/expression of tumor suppressor genes, namely the forkhead boxo3 (also known as Foxo3), a transcription factor encoded by the Foxo3 gene. Foxo3 plays pivotal roles in tumorigenesis and drug resistance. Thus, Foxo3 deregulation appears to be essential in the development of cancer.

**Figure 7:**
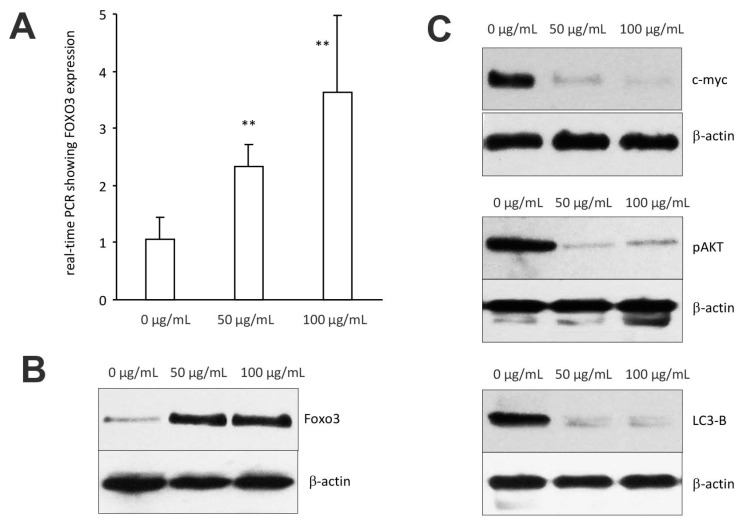
Expression of signal proteins affected by *C. coccineum:* (**A**) MDA-MB-231 cells were cultured to subconfluency and treated with ethanol extract of *C. coccineum* (50 μg/mL). Total RNAs were harvested followed by measurement of Foxo3 expression by real-time PCR. Treatment with *C. coccineum* increased Foxo3 mRNA expression. (**B**) Cell lysate was also prepared from the cells treated with *C. coccineum* followed by Western blotting probing with antiFoxo3 antibody. Similarly, treatment with *C. coccineum* increased Foxo3 protein expression. (**C**) Expression of c-myc, pAKT, and LC3-B was also analyzed in the cell lysate. Treatment with *C. coccineum* decreased expression of these proteins. ** *p* < 0.01.

In the cells treated with 50 μg/mL of ethanol extract of *C. coccineum*, Foxo3 expression was measured by real-time PCR using total RNAs isolated from MDA-MB-231 cells treated with or without the extract, using the primers listed in the Methods section. We found that Foxo3 mRNA expression was significantly upregulated as compared with the control (Figure 7A). Western blot analysis revealed that Foxo3 protein also increased in the cells treated with *C. coccineum* ethanol extract (Figure 7B).

We also examined expression of other proteins that are associated with Foxo3-induced cancer cell death including c-myc, pAKT, and LC3-B. Treatments with *C. coccineum* at the concentrations of 50 μg/mL and 100 μg/mL greatly decreased expression of these proteins (Figure 7C), suggesting a role in the inhibition of tumor growth.

These results provide new insight for future development of *Cynomorium* plants as an effective and safe medicinal plant for use by cancer patients. It also provides evidence allowing us to further understand the molecular mechanism by which *C. coccineum* exerts its anticancer activity. Amongst all medicinal plants, *C. coccineum* and *C. songaricum* are two popular plants used in Asian countries and in the Arab world, especially for reproductive activity. These two plants have been widely used for the promotion of health longevity and immunomodulatory effects. We found that the anticancer activity of both *C. coccineum* and *C. songaricum* was significantly higher than that of the three well-known medicinal mushrooms (*Amauroderma rude*, *Ganoderma lucidum*, and *Coriolus vesicolor*). In our previous study, we found that *Amauroderma rude* possessed the highest anticancer activity in inducing cell death, tested in several invasive and metastatic breast cancer cell lines and other types of cancer cell lines [30]. The anticancer activities of components isolated from *C. coccineum*, and *C. songaricum* were not only better than *Amauroderma rude*, but also Ergosterol purified from *Ganoderma lucidum*, which also possessed extremely high anticancer activity. Finally, the anticancer effects of *C. coccineum* and *C. songaricum* were significantly stronger than the anticancer drugs Vinblastine. Our results suggest that understanding the biology and physiological roles of *C. coccineum* will soon allow this plant to be used as an adjuvant for health promotion, especially in cancer patients. Furthermore, the extracts of *C. coccineum* can be regarded as a valuable adjuvant for combination chemotherapy in the treatment of various cancers. Future work could lead to the purification and synthesis of the main active molecules from *Cynomorium* plants to improve its effective therapy in clinical settings.

## 4. Conclusions

In vitro, *C. coccineum* possesses the highest activity in killing breast cancer cells. In vivo, *C. coccineum* inhibits cancer progression and prolongs mouse survival. *C. coccineum* induces up-regulation expression of the cancer suppressor Foxo3 and its related molecules in the signaling pathway.

## Abbreviations

DMEM	Dulbecco’s modified Eagle’s medium:
FBS	fetal bovine serum;
PAGE	Polyacrylamide gel electrophoresis.
